# Clinical and Neuroimaging Features in a Patient with Non-Ketotic Hyperglycemia

**DOI:** 10.3390/neurolint12030018

**Published:** 2020-12-14

**Authors:** Yonghua Zhang, Aasheeta Parikh

**Affiliations:** Edward Neuroscience Institute in Affiliation with Northwestern Medicine, 801 S. Washington Street, Naperville, IL 60540, USA; Aasheeta.Bawa@eehealth.org

**Keywords:** non-ketotic hyperglycemia, hemichorea–hemiballism, neuroimaging

## Abstract

Hemichorea–hemiballism (HC–HB) is a spectrum of involuntary flinging and flailing, non-patterned, irregular movements involving one side of the body. A rare dysfunction of glucose metabolism leading to a state of non-ketotic hyperglycemia (NKH) is thought to be a cause of these symptoms. In previous case studies, imaging findings have been in the basal ganglia as hyperintense lesions on magnetic resonance imaging (MRI) or hyperdensities on computerized tomography (CT). This case is unique due to abnormal findings in the MRI T2/fluid-attenuated inversion recovery (FLAIR) sequence in areas not previously reported—the thalamus and midbrain/pons. As in other NKH cases, the patient improved both clinically and radiologically. In patients with uncontrolled diabetes and abnormal movements, monitoring of blood glucose is imperative as it can lead to recognition of HC–HB. Other etiologies, including stroke, neoplasm, demyelination, and inflammatory processes, have uncertain prognoses with unfavorable outcomes. The prognosis for NKH is usually favorable, and thus important to identify.

## 1. Introduction

The patient presented with altered mental status, left-sided weakness, dysarthria, hemichorea–hemiballism (HC–HB), disinhibited behavior, and abnormal brain imaging. It was initially thought that he had a stroke, but subsequent imaging ruled out stroke and was more concerning for malignant neoplasm or demyelinating disease. Over four months, his clinical symptoms resolved completely, and subsequent imaging normalized as well, and he was retrospectively diagnosed with non-ketotic hyperglycemia (NKH). His clinical outcome is excellent, with no residual neurological deficits. These clinical symptoms often mimic those of more malignant processes including stroke, seizure, neoplasm, demyelinating disease, infection, and thus differentiating studies such as computerized tomography (CT), magnetic resonance imaging (MRI), cerebrospinal fluid (CSF) studies, and electroencephalography (EEG) are crucial.

## 2. Case Report

A 57-year-old Chinese male presented to the emergency department in January 2017 due to acute onset left facial droop, left arm weakness, slurred speech, and dizziness. His past medical history included hypertension, diabetes mellitus type 2, Hashimoto disease, and colon polyps. His initial presentation was most consistent with ischemic stroke, though he was not a candidate for alteplase or neuro-intervention due to a low National Institutes of Health Stroke Scale (NIHSS) score and the fact that he fell outside the window for these treatments. His exam was significant for left hemichorea and hemiballismus movements, left-sided weakness, and ataxia. There were no significant abnormalities upon reviewing vital signs. The main laboratory abnormalities were hemoglobin A1c of 8.7% and elevated glucose in cerebrospinal fluid (CSF) of 97. The remainder of CSF was normal or negative, including protein, cell count, Lyme titer, oligoclonal bands, myelin basic protein, immunoglobulin G, albumin, and culture. Serum workup, including antinuclear antibody, erythrocyte sedimentation rate, lactate dehydrogenase, comprehensive metabolic panel, and complete blood count, was unremarkable. After an initial work up of imaging and labs, steroids were empirically started. He unfortunately promptly developed behavioral changes, including disinhibition, impulsivity, irritability, and inappropriate behavior. Eventually, he was tapered off steroids with an improvement in behavioral issues. He remained in state of non-ketotic hyperglycemia with an average serum glucose of 314 mg/dL (glucose range 189–440 mg/dL) during the two-week admission. This case report intended for medical/educational activity is considered not research by the DHHS (Department of Health and Human Services) definition therefore an Institutional Review Board approval is not required. The patient provided their written informed consent for the publication of the case report.

Diagnosis remained elusive, in part due to neuro-imaging findings with many possible etiologies. An initial head CT was normal, and non-contrast MRI was negative for stroke but showed a possible mass with edema involving the right thalamus, posterior limb of the right internal capsule, right pons, and right cerebral peduncle. MRI with contrast revealed a 1.9 cm region of lobulated enhancement centered within the right paracentral midbrain and anterior aspect of the right cerebral peduncle with a surrounding T2/fluid-attenuated inversion recovery (FLAIR) hyperintense signal which was concerning with regard to a primary glial neoplasm such as an astrocytoma with lymphoma or a demyelinating/inflammatory process. MRI Brain Spectroscopy (MRS) demonstrated the mild elevation of choline in relation to creatine. There was also the suggestion of some necrosis with mildly elevated lactate peaks, suggestive of primary glioma, but we could not exclude the possibility of an inflammatory process. Repeated brain MRIs and repeat head CTs were unchanged, demonstrating restricted diffusion, T2/FLAIR hyperintensity, enhancement, and edema. Overall, the imaging findings remained non-specific. A chest/abdomen/pelvis CT showed no enlarged adenopathy in the chest, abdomen or pelvis, and multiple low attenuation lesions in the liver, which were not malignant in appearance. 

Differentials included adverse drug effects, Huntington’s disease, stroke, alcoholic-induced liver disease, lymphoma, demyelinating disease, and an intermediate grade primary glial neoplasm. Imaging patterns and the response to steroids suggested a demyelinating process versus a central nervous system neoplasm, but the lesion’s location made biopsy dangerous. The case was discussed in the hospital’s multi-disciplinary tumor conference, with input from neuroradiologists, neurosurgeons from Northwestern University, oncology, and radiation–oncology. The diagnostic uncertainty was troubling to the team; however, since biopsy was unsafe, a working diagnosis of high-grade glioma was given. He was discharged with plan to start radiotherapy in 6–8 weeks, but due to rapid improvement on repeat MRI in March 2017, he did not begin treatment. He also returned to the neurology clinic for a follow-up in March 2017, and his exam was pertinent for the resolution of HC–HB. Left hand coordination remained poor, and he continued to display mild frontal lobe disinhibition at that time, though to a much lesser degree. In May 2017, he had a repeat MRI with a nearly complete resolution of previous FLAIR abnormality and an intact neurological exam. 

**Figure 1:**
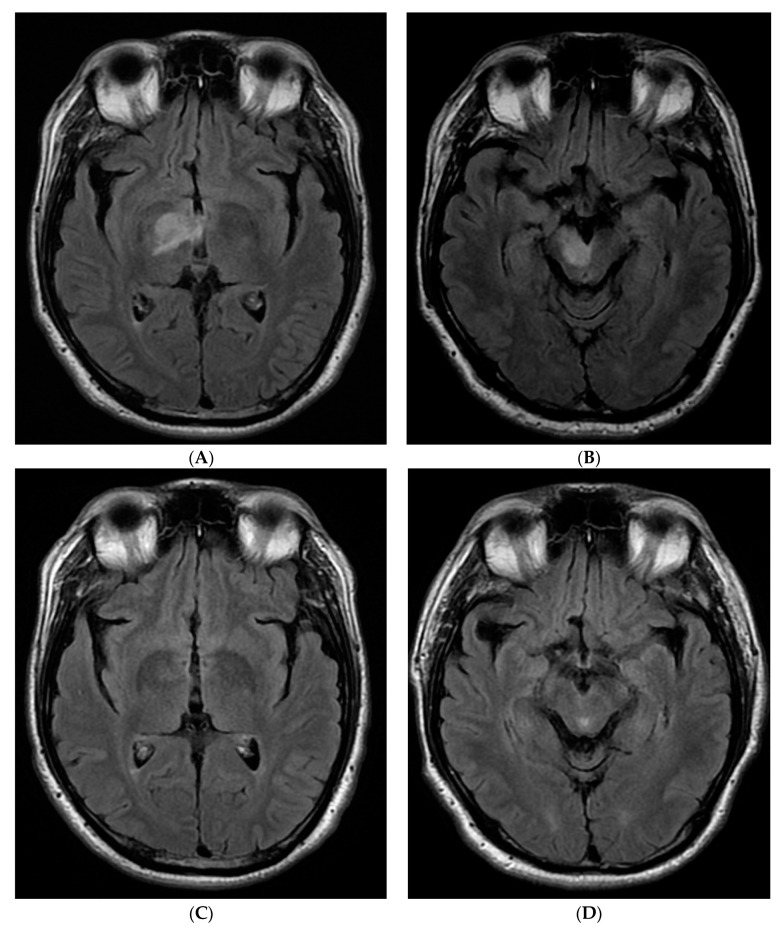
Selected magnetic resonance imaging (MRI) T2/fluid-attenuated inversion recovery (FLAIR) imaging from initial (**A**,**B** from Jan 2017) and last (**C**,**D** from May 2017) MRI.

**Figure 2:**
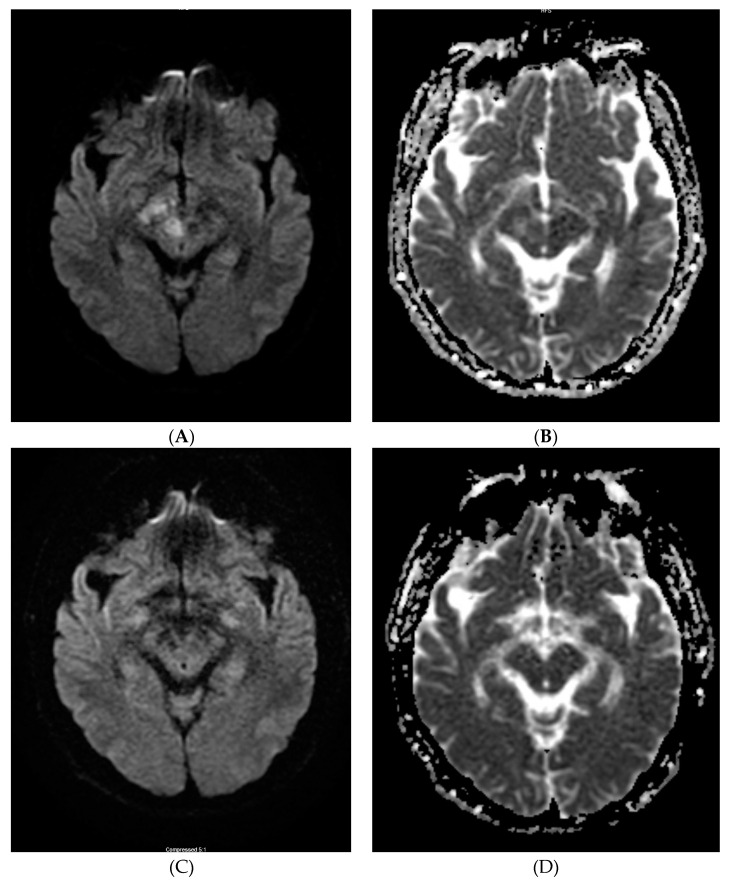
Selected MRI diffusion-weighted imaging (DWI)/apparent diffusion coefficient (ADC) imaging from initial (**A**,**B** from Jan 2017) and last (**C**,**D** from May 2017) MRI.

A series of repeat brain MRIs over 1–4 months showed significantly decreased FLAR abnormalities and decreased nodular enhancement in T2/FLAIR and, eventually, the complete resolution of FLAIR abnormalities and enhancement (Figure 1) as well as restriction diffusion from diffusion-weighted imaging (DWI)/apparent diffusion coefficient (ADC) (Figure 2).

## 3. Discussion

Hemichorea–hemiballism (HC–HB) is a spectrum of involuntary, continuous, non-patterned movements involving one side of the body [1], and can be a rare and debilitating presentation of hyperglycemia [2]. It is thought to be caused by the dysfunction of glucose metabolism. Brain imaging abnormalities have commonly been reported as MRI T1 hyperintense lesions or CT hyperdensities in the basal ganglia. Since asymmetric MRI hyperintensities in T2/FLAIR with the involvement of the unilateral thalamus, extending into the cerebellar peduncle and mid brain/pontine, have never been reported, these authors present this case—unique in its neuroimaging sequence features, anatomical location, and confounding initial diagnosis of malignant brain mass. Clinical improvement was noted before initial imaging began improving, although the lesions eventually regressed and resolved.

Neuroimaging findings in non-ketotic hyperglycemic patients with HC–HB are usually unilateral basal ganglia hyperdensity on CT or on T1W MRI, contralateral to the symptomatic side [3,4,5,6,7,8]. On T2 MRI, the signal changes from the lesions can be hypointense, isointense, or hyperintense [3,9], but are more predominantly hypointense. The pathogenesis of these imaging findings is unclear. Some possible mechanisms include: 1. gemistrocyte accumulation (gemistocytes are swollen astrocytes which contain rich protein; they initially appear during acute injury but later gradually shrink); 2. hyperviscosity (hypervicosity is responsible for the restriction diffusion in HC–HB caused by hyperglycemia [10]); 3. neuronal dysfunction and possible cytotoxic edema (which may potentially be responsible for MRI DWI/ADC changes in Figure 2 in this case). A combination of these are likely responsible for the observed MRI imaging abnormalities in susceptible diabetic individuals [10]. Different studies have obtained variable histopathological results [11,12]. One theory that focal hemorrhage and calcification lead to abnormal brain imaging was not supported by histopathology, nor by the fact that most imaging abnormalities have complete resolution. A possible mechanism for the resolution of MRI T2/FLAIR findings may be due to reversible ischemic insult potentiated by hyperglycemia [13]. However, the mechanisms causing brain imaging findings associated with NKH remain controversial.

There are also studies which have reviewed the MR spectroscopy (MRS), although results are somewhat variable. The ratio between choline (Cho) and creatinine (Cr) were significantly higher in patients with HC–HB lesions [13] In a small study, patients had a reduced ratio of N-acetylaspartate/creatine (NAA/Cr), the elevation of the Cho/Cr ratio, and the presence of a lactate peak (the ratios were normal in the contralateral hemisphere and controls) [14]. Our patient also had mild elevation of Cho/Cr and mildly elevated lactate peaks. Low NAA/Cr on MRS was also reported by Roy et al. [15].

Moreover, it is important to note that our patient is of Asian origin. Two cases of NKH associated with an abnormal MRI signal reported by Wintermark et al. [16] and 30 out of 35 cases analyzed by Lin et al. [17] were described in Asian patients, suggesting a genetic disposition. Oh S-H et al. [5] reported that HC–HB from NKH is found to be more common in Asian descendants, again suggestive of a probable element of genetic predisposition. However, in a case report with two patients by Yacoub HA in 2013 [18], neither of the patients were Asian. Further investigations into a potential ethnic link may occur as more cases are reported.

## 4. Conclusions

In patients with uncontrolled diabetes and abnormal movements, the monitoring of blood glucose is vital, as it can lead to the recognition of HC–HB. MRI may reveal hyperintensities in the basal ganglia, thalamus and midbrain/pons. Although rare, this dysfunction of glucose metabolism can cause neurological symptoms. Unlike other etiologies, including stroke, neoplasm, demyelination, and inflammatory processes, whose prognoses are more uncertain and more likely to have worse outcomes, the prognosis for NKH is usually favorable.

## Abbreviations

NKH	non-ketotic hyperglycemic
HC–HB	hemichorea and hemiballism
CSF	cerebrospinal fluid
